# NG2 proteoglycan promotes tumor vascularization via integrin-dependent effects on pericyte function

**DOI:** 10.1007/s10456-013-9378-1

**Published:** 2013-08-08

**Authors:** Weon-Kyoo You, Fusanori Yotsumoto, Kenji Sakimura, Ralf H. Adams, William B. Stallcup

**Affiliations:** 1 Tumor Microenvironment and Metastasis Program, Sanford-Burnham Medical Research Institute, Cancer Center, 10901 North Torrey Pines Road, La Jolla, CA 92037 USA; 2 Present Address: Biologics Business, Research and Development Center, Hanwha Chemical, 76 Gajeong-Ro Yuseong-Gu, Daejeon, 305-804 South Korea; 3 Department of Cellular Neurobiology, Brain Research Institute, Niigata University, Niigata, 951-8585 Japan; 4 Department of Tissue Morphogenesis, Max Planck Institute for Molecular Biomedicine and University of Muenster, Roentgenstrasse 20, 48149 Muenster, Germany

**Keywords:** Blood vessels, Co-culture systems, Endothelial cells, NG2 proteoglycan, Pericytes, β1 integrins

## Abstract

The NG2 proteoglycan stimulates the proliferation and migration of various immature cell types, including pericytes. However, the role of NG2 in mediating pericyte/endothelial cell interaction has been less clear. In this study, we show that pericyte-specific NG2 ablation causes several structural deficits in blood vessels in intracranial B16F10 melanomas, including decreased pericyte ensheathment of endothelial cells, diminished formation of endothelial junctions, and reduced assembly of the vascular basal lamina. These deficits result in decreased tumor vessel patency, increased vessel leakiness, and increased intratumoral hypoxia. NG2-dependent mechanisms of pericyte interaction with endothelial cells are further explored in pericyte/endothelial cell co-cultures. siRNA-mediated NG2 knockdown in pericytes leads to reduced formation of pericyte/endothelial networks, reduced formation of ZO-1 positive endothelial cell junctions, and increased permeability of endothelial cell monolayers. We also show that NG2 knockdown results in loss of β1 integrin activation in endothelial cells, revealing a mechanism for NG2-dependent cross talk between pericytes and endothelial cells.

**Electronic supplementary material:**

The online version of this article (doi:10.1007/s10456-013-9378-1) contains supplementary material, which is available to authorized users.

## Introduction

In addition to its importance in normal development, the process of neovascularization is also a critical aspect of many pathologies, including tumor growth, rheumatoid arthritis, systemic lupus erythematosus, psoriasis, macular degeneration, and proliferative retinopathy [1]. Neovascularization involves active expansion and remodeling of capillaries and arterioles, which represent the microvessel equivalents of arteries. These structures are composed of three major elements: endothelial cells, pericytes, and the vascular basement membrane. Interactions and signaling between these three components are essential for microvessel development during both developmental and pathological neovascularization [2–4]. Changes in pericyte and endothelial cell recruitment, abnormalities in pericyte/endothelial cell interaction, or defects in assembly of the vascular basement membrane can all lead to alterations in cross talk among microvessel components that affect vessel morphogenesis, maturation, and function [2, 4–6]. Identifying key molecular components of microvessels and elucidating the respective roles of these molecules in mediating interactions between microvascular elements is therefore essential for our ability to understand deficits in cell communication that underlie microvessel dysfunction.

A major topic of study in our laboratory has been the role of pericytes in microvessel morphogenesis and function, with a special focus on the participation of the NG2 chondroitin sulfate proteoglycan in these processes. NG2 is a prominent component of activated pericytes, but not endothelial cells, in both normal and pathological microvessels. The proteoglycan not only serves as one of the most reliable pericyte markers [7, 8], but also plays important roles in pericyte recruitment and interaction with endothelial cells during microvessel development [9–13]. Germline ablation of NG2 in the mouse leads to deficits in pathological retinal and corneal vascularization [12], as well as to deficits in tumor vascularization that correlate with slower progression of both engrafted B16F10 melanomas in the brain [11] and spontaneous MMTV-PyMT mammary tumors [10]. Tumor vessels in the NG2 null mouse are characterized by reduced pericyte coverage of endothelial cells and by diminished assembly of the vascular basement membrane [10, 11]. Altered interactions between these key microvessel components lead to deficits in both pericyte and endothelial cell maturation [6], and these changes at the cellular and structural levels lead to decreased tumor vessel patency and increased tumor vessel leakiness. Overall, tumors in NG2 null mice exhibit increased levels of hypoxia, due at least in part to poor vessel function [10, 11].

These experimental results strongly suggest that NG2 is a key molecule in mediating cross talk between pericytes and endothelial cells. However, our interpretation of the results is limited by the fact that NG2 is expressed in the tumor stroma not only by microvascular pericytes, but also by other stromal cell types. For example, NG2 is transiently expressed by macrophages in most, if not all, solid tumors. In the case of mammary tumors, NG2 is also expressed by adipocytes, and in brain tumors, the proteoglycan is expressed by oligodendrocyte progenitor cells. Therefore, in addition to the effects of NG2 ablation on pericyte function, it is possible that the absence of NG2 from other stromal cell types can affect their production of factors that contribute to tumor vascularization and progression. In order to examine the pericyte-specific effects of NG2 ablation on vascularization, we have utilized both in vivo and in vitro models to study the effects of diminished NG2 expression on microvessel formation. In vivo, we have used Cre/lox technology to create pericyte-specific NG2 null mice in which we examine the properties of microvessels in engrafted intracranial B16F10 melanomas. Tumor vessels in these mice exhibit many of the same deficits seen in tumor vessels in germline NG2 null mice. In vitro, we have used siRNA technology to knock down NG2 expression in human microvascular pericytes, followed by examination of the ability of these cells to interact with human endothelial cells in the assembly of vascular networks. Compared with co-cultures of endothelial cells and control pericytes, network formation is significantly reduced in the case of NG2 knockdown pericytes. Use of a conformationally sensitive β1 integrin antibody has allowed us to demonstrate that NG2 knockdown in pericytes leads to reduced β1 integrin signaling in pericytes that correlates with loss of proliferation and motility. In addition, pericyte-dependent β1 integrin signaling in closely apposed endothelial cells is reduced by NG2 knockdown in pericytes, providing a possible explanation for the impaired morphogenesis seen in assays of network formation. Decreased β1 integrin signaling in endothelial cells also results in reduced formation of ZO-1-positive intercellular junctions, leading to diminished barrier function of endothelial monolayers. These results reinforce the conclusion that NG2 expression by pericytes is important not only for pericyte biology, but also for pericyte interactions with endothelial cells.

## Materials and methods

### Cell lines

Human brain microvascular pericytes (ScienCell) and human umbilical vein endothelial cells (HUVECs, Lonza) were maintained, respectively, in pericyte medium (PM, ScienCell) and endothelial basal medium-2 (EBM-2, Lonza) containing appropriate growth supplements. NG2-negative B16F10 melanoma cells [14] were maintained in DMEM containing 10 % fetal bovine serum.

### Animals and tumor implantation

Mice were maintained in the Sanford-Burnham Vivarium (fully accredited by the Association for Assessment and Accreditation of Laboratory Animal Care). All animal procedures were performed in accordance with Office of Laboratory Animal Welfare regulations and were approved by Sanford-Burnham Institutional Animal Care and Use Committee review prior to execution. NG2 floxed mice [15] and pdgfrb-Cre transgenic mice [16, 17] were backcrossed to C57Bl/6 backgrounds for more than 10 generations.

Intracranial B16F10 tumors were established as previously described [6, 11]. Tumors were analyzed at 7 days post injection. Pericyte/endothelial cell overlap, basement membrane assembly, vessel patency, leakage, and intratumoral hypoxia were assessed by previously described methods [10, 11].

### Immunohistochemistry and microscopy

Pericytes, endothelial cells, and apoptotic cells were identified by staining with combinations of 2 or 3 antibodies. Pericytes were labeled with rabbit anti-NG2, guinea pig anti-NG2, or rabbit anti-PDGFRβ (1:100, [6, 11]). Endothelial cells were labeled with rabbit anti-human CD31 (1:500; Abcam) or anti-human CD31 (1:500; BD Pharmingen) for HUVECs in culture and with hamster anti-mouse CD31 (1:500; Pierce) or rat anti-mouse CD31 (1:500; BD Pharmingen) in the case of mouse tumors. Endothelial cell junctions in mouse tumor vessels were identified by labeling for ZO-1 (1:500; Invitrogen). Macrophages were labeled by rat anti-F4/80 (1:500, Invitrogen) antibody. Vascular basement membrane assembly was assessed by labeling for collagen IV (1:500, Millipore). Apoptotic cells were labeled with rabbit antibody against activated caspase-3 (1:500; R&D Systems), and pericyte proliferation was assessed by staining for phosphohistone H3 (1:500, Cell signaling). β1 integrin activation was assessed by labeling with monoclonal anti-human integrin β1 (HUTS-21, 1:50, BD Pharmingen) as described in previous studies [9, 18–20]. Activation of focal adhesion kinase was assessed by phospho-FAK (1:500, Invitrogen) staining. Total integrin β1 was labeled by monoclonal anti-integrin β1 (TS2/16, 1:100, American Type Culture Collection). Secondary antibodies included FITC-, Cy3-, or Cy5-labeled (1:400; Jackson ImmunoResearch Laboratories Inc.), and Alexa 488- or Alexa 568-labeled (1:250; Invitrogen) goat, donkey, or mouse anti-rat, anti-hamster, anti-rabbit, anti-goat, or anti-mouse IgG. Examination and image capture (TIFF images) from immunostained tumor sections and from in vitro cell cultures were accomplished using a Fluoview 1000 (Olympus) Laser Point Scanning Confocal Microscope and an Inverted TE300 Nikon Fluorescence Microscope as described previously [611]. Areas (number of pixels) with immunostaining greater than a set threshold were quantified using computer-based morphometry software (Image-Pro Plus 4.5, Media Cybernetics, Inc.).

### NG2 downregulation by siRNA transfection

Expression of NG2 proteoglycan by human brain microvascular pericytes was downregulated by transfection of siRNA targeted to NG2 (GCUAUUUAACAUGGUGCUGtt, siRNA ID#: 146147, Ambion). Downregulation of NG2 expression was quantified by immunocytochemistry.

### Cell proliferation and migration

Seventy-two hours after siRNA transfection, pericyte numbers were measured by determining the density of DAPI-positive nuclei per defined area. Migration of pericytes was examined in 24-well transwell plates (8.0-μm pore size, Costar) via published methods [9].

### Vascular network formation in pericyte/endothelial cell co-cultures

Vascular network formation in three dimensions was studied by co-culturing HUVECs and pericytes in Matrigel [21]. Pericytes were pre-treated with NG2-targeting siRNA, scrambled siRNA, or GAPDH-targeting siRNA. Matrigel (BD 356231, growth factor reduced, phenol red-free) was loaded (0.25 ml) in chilled 4-well CultureSlides (354114, BD) or (0.1 ml) glass bottom microwell dishes (P35G-1.5-14-C, MatTek), and allowed to gel at 37 °C for 8 h. For fluorescence imaging, pericytes and HUVECs were pre-stained with 4 μM CellTracker Red CMTPX (Invitrogen) and CellTracker Green CMFDA (5-chloromethylfluorescein diacetate, Invitrogen), respectively, in basal medium for 30 min, and then incubated overnight in either PM or EBM. Labeled pericytes and HUVECs were harvested by trypsinization, resuspended in EBM-2 medium (5 × 10^4^ cells/ml), and mixed at a 1:4 pericyte/HUVEC ratio. This mixture (0.5 ml or 2.5 ml) was seeded in 4-well CultureSlides or glass bottom microwell dishes, respectively. HUVEC/pericyte networks began to form after 2–4 h. Network formation was evaluated by scanning confocal microscopy.

### Endothelial cell permeability

Permeability of endothelial cell monolayers in vitro was studied in 24-well transwell plates (0.4-μm pore size, Costar) using in-contact co-cultures of pericytes and HUVECs [22, 23] established on opposite sides of transwell inserts. Endothelial cells were seeded on the upper surface of the membrane at a density of 10^5^ cells/ml, while pericytes were seeded on the lower surface of the membrane at a density of 5 × 10^4^ cells/ml. For non-contact co-cultures, pericytes were seeded on the bottom of the transwell rather than on the lower surface of the membrane. In some cases, endothelial cell monolayers were used in the absence of pericyte monolayers (see Fig. 6a). Medium in the lower chamber contained VEGF (5 ng/ml), and medium in the upper chamber contained FITC-dextran (0.2 mg/ml, 250 kDa, Sigma) and VEGF (5 ng/ml). After 5 h of incubation, the FITC-dextran content of lower chambers was determined by absorbance at 494 nm. Purified, soluble, recombinant NG2 used in these experiments was prepared according to [9, 24].

### Statistical analysis

All results are expressed as mean ± SE. Statistical analyses were performed using the two-tailed *t* test. *P* < 0.05 was considered statistically significant.

## Results

### Pericyte-specific ablation of NG2 leads to deficits in tumor blood vessel structure and function

**Fig. 1:**
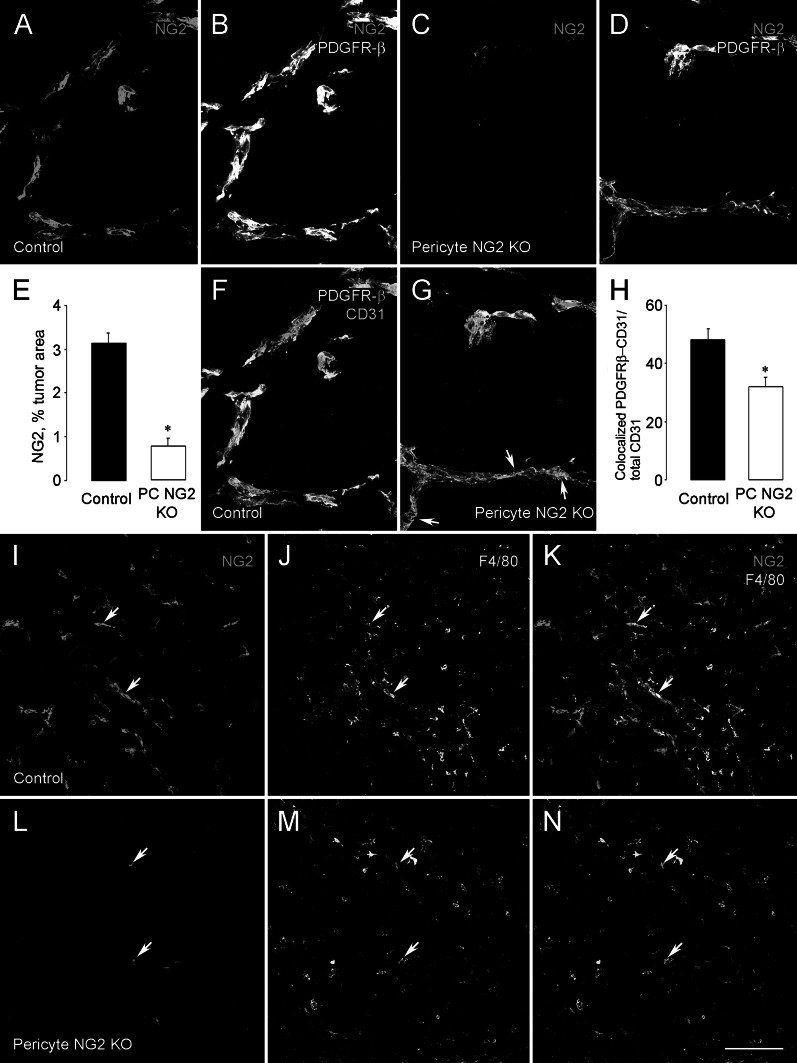
Pericyte-specific NG2 ablation leads to reduced pericyte ensheathment of endothelial cells. NG2 proteoglycan (*red*) is co-expressed with PDGFRβ (*green*) in pericytes in B16F10 tumor vessels in control mice (**a**, **b**). However, in tumors in pericyte-NG2ko mice, pericyte expression of NG2 is largely abolished (**c**, **d**). Total NG2 expression (NG2-positive pixels) is reduced by 75 % in tumors in pericyte-NG2ko mice (**e**). Pericyte ensheathment of endothelial cells (**f**, **g**) was evaluated by double staining for PDGFRβ (*green*) and CD31 (*blue*), allowing determination of PDGFRβ/CD31 overlap in *z*-stacks of confocal images. Overlap of PDGFRβ pixels with CD31 pixels is reduced in pericyte-NG2ko tumor blood vessels (*arrows* in **g**). Quantification reveals a 31 % decrease in pericyte ensheathment of endothelial cells (**h**). Macrophages, identified by immunostaining for F4/80 (*green*), contribute to the overall level of NG2 expression (*red*) in tumors in control mice (**i**–**k**, *arrows*). In tumors in pericyte-NG2ko mice, NG2 expression is retained by macrophages (**l**–**n**, *arrows*), but not by pericytes. *P < 0.05 versus control mice. *Scale bar* 40 μm (**a**–**d**, **f**, **g**), 120 μm (**i**–**n**). (Color figure online)

NG2 ^flox/flox^ females were crossed with NG2 ^flox/wt/pdgfrb-Cre^ males to generate NG2 ^flox/flox^ (control) and NG2 ^flox/flox/pdgfrb-Cre^ (pericyte-NG2ko) progeny. B16F10 melanomas were established in the brains of these two sets of mice. In tumors from pericyte-NG2ko mice, we confirmed that NG2 expression is absent from pericytes (Fig. 1). Whereas in control mice, NG2 is strongly expressed by PDGFRβ-positive pericytes (Fig. 1a, b), in pericyte-NG2ko mice, NG2 is largely undetectable in these cells (Fig. 1c, d). Quantification of NG2-positive pixels reveals a 75 % reduction in NG2 expression in tumors from the pericyte-NG2ko mice (Fig. 1e). Although NG2 is not expressed by the B16F10 melanoma cells [11], it is expressed by macrophages [10]. We therefore confirmed the pericyte-specific ablation of NG2 via immunostaining for the macrophage marker F4/80. In both control and pericyte-NG2ko mice, we were able to identify F4/80-positive macrophages that expressed NG2 (Fig. 1i–n, arrows). Since pericytes are clearly NG2-negative in pericyte-NG2ko mice, NG2 expression by macrophages at least partially accounts for residual NG2 expression in these mice.

**Fig. 2:**
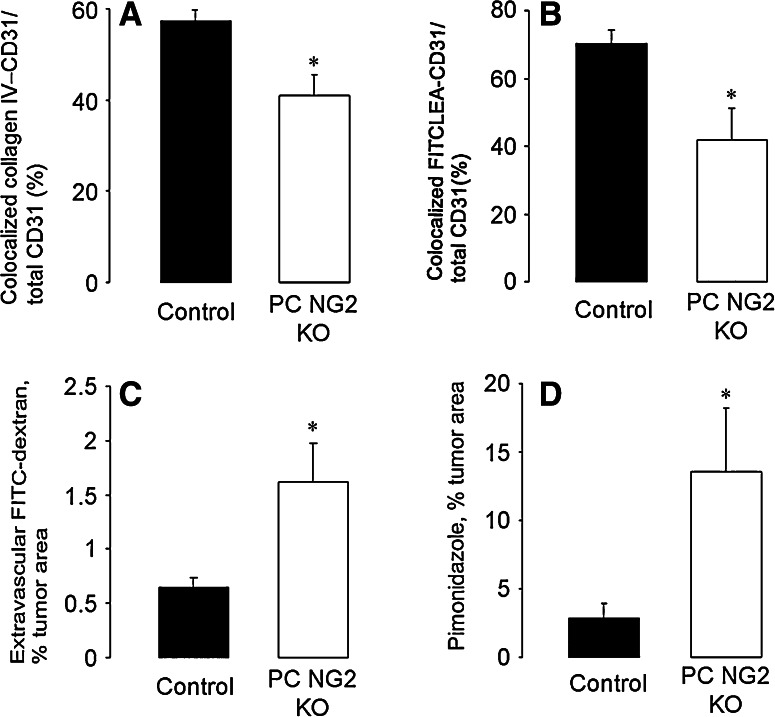
Pericyte-specific NG2 ablation leads to structural and functional deficits in tumor blood vessels. Vascular basal lamina deposition, assessed by staining for type IV collagen associated with the CD31-positive vascular endothelium, is significantly reduced in tumor blood vessels of pericyte-NG2ko mice (PC NG2 KO; panel **a**, 25 % reduction). Tumor vessel patency was evaluated by perfusion of animals with FITC-LEA, followed by immunostaining of sections for CD31. Functional vessels are labeled for both FITC-LEA and CD31, while non-functional vessels are labeled only for CD31. Patency is reduced by 40 % in tumor vessels in pericyte-NG2ko mice (panel **b**). Vessel leakiness was assessed by perfusion of animals with FITC-dextran, followed by immunostaining of sections for CD31. Leakage is quantified by measuring FITC-dextran located outside of CD31-positive blood vessels. Vessels in pericyte-NG2ko mice are almost threefold leakier than vessels in control mice (panel **c**). Intratumoral hypoxia was measured after perfusion of animals with pimonidazole hypoxia probe and immunostaining of sections with antibody against pimonidazole. Areas of hypoxia are increased almost sixfold in pericyte-NG2ko mice (panel **d**). *P < 0.05 versus control mice. Experimental details of these analyses can be found in [10, 11]

We also were able to determine that ensheathment of CD31-positive endothelial cells by PDGFRβ-positive pericytes is reduced in tumor vessels in pericyte-NG2ko mice (Fig. 1f, g). Compared to control mice, endothelial cells are less well covered by pericytes in pericyte-NG2ko mice (arrows in G). Quantification of coverage reveals a 30 % decrease in pericyte ensheathment in the absence of pericyte NG2 (Fig. 1h). Similar to our previous findings with tumor blood vessels in germline NG2 knockout mice [10, 11], this NG2-dependent decrease in pericyte/endothelial cell interaction leads to additional structural deficits in the vasculature. In particular, we noted diminished assembly of the vascular basal lamina, as revealed by determining the overlap between CD31 labeling and labeling for the basement membrane marker collagen IV. Quantification of these data demonstrates a 30 % decrease in basal lamina assembly in pericyte-NG2ko mice (Fig. 2a). In addition, we used comparisons of CD31 staining (all endothelial cells) and perfused FITC-LEA staining (endothelial cells in functional vessels) to determine that functional tumor vessels (vessel patency) are reduced by 40 % in pericyte-NG2ko mice (Fig. 2b). Structural deficits in pericyte-NG2ko tumor vessels also resulted in roughly a threefold increase in leakage of perfused FITC-dextran into the extravascular space (Fig. 2c). These diminished functional properties of pericyte-NG2ko vessels are reflected by a sixfold increase in intratumoral hypoxia, as determined by quantifying retention of the hypoxia probe pimonidazole (Fig. 2d).

### NG2 knockdown inhibits pericyte proliferation and migration in vitro

**Fig. 3:**
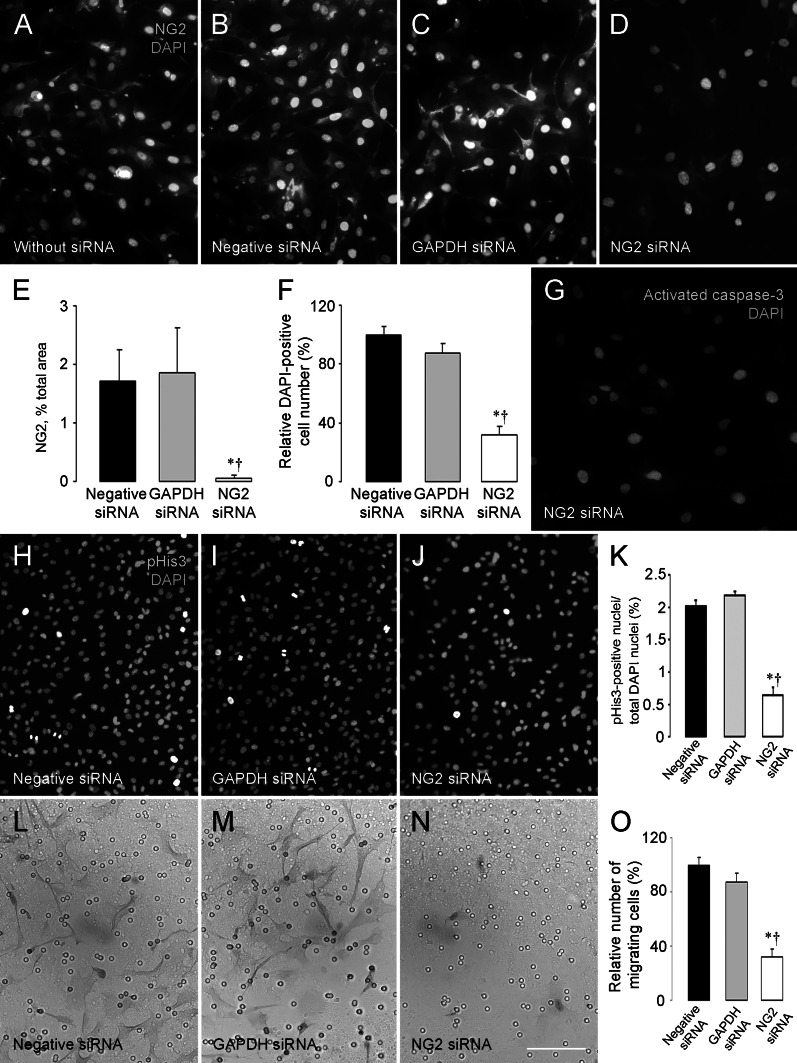
NG2 downregulation inhibits pericyte proliferation and migration. NG2 expression (*red*) by human brain microvascular pericytes is not affected by treatment with negative siRNA (**b**) or GAPDH siRNA (**c**), compared to NG2 expression by control pericytes (**a**). However, NG2 expression is largely abolished by treatment with NG2-targeting siRNA (**d**). NG2-positive pixels are reduced by 98 % in pericytes treated with NG2 siRNA (**e**). In addition, the number of DAPI-positive pericyte nuclei (*blue*) is markedly lower (64–70 % reduction) in NG2 siRNA-treated cultures compared to control cultures (**f**), suggestive of reduced proliferation in the absence of NG2. Immunostaining for activated caspase-3 (*red*) does not reveal any evidence of apoptosis in NG2 siRNA-treated cultures (**g**). However, immunostaining for phosphohistone H3 (*red*) demonstrates a 70 % reduction in mitotic index in NG2 siRNA-treated cultures compared to control siRNA-treated cultures (**h**–**k**). Pericytes treated with negative siRNA (**l**) or GAPDH siRNA (**m**) actively migrate through 8-μm pores (*round circles*) in transwell membranes in response to stimulation with PDGF-BB (20 ng/ml). However, migration of NG2 siRNA-treated pericytes is markedly reduced under these conditions (**n**, **o**). Pericytes are stained with hematoxylin. *Blue* = DAPI. *P < 0.05 versus negative siRNA, ^†^ *P* < 0.05 versus GAPDH siRNA. *Scale bar* 120 μm (**a**–**d**, **g**, **l**–**n**), 240 μm (**h**–**j**). (Color figure online)

In order to elucidate mechanisms that underlie the vascular deficits observed in tumor vessels in pericyte-NG2ko mice, we used siRNA methodology to knock down NG2 expression in pericytes in vitro. Compared to transfection with control siRNA species (Fig. 3a–c), transfection with NG2-targeted siRNA results in virtually complete loss (more than 98 %) of NG2 expression by human brain microvascular pericytes (Fig. 3d, e). Since NG2 expression is often associated with enhanced cell proliferation and motility [13], we compared these properties in control pericytes and pericytes treated with NG2 siRNA. After siRNA treatment, pericyte numbers in the NG2 siRNA-treated population are reduced almost threefold compared to numbers of control siRNA-treated pericytes (Fig. 3a–d, f). Reduced pericyte numbers could also be due to increased pericyte apoptosis following NG2 ablation [18, 20], although we did not detect morphological signs of pericyte apoptosis in this population. Labeling for activated caspase-3, a more sensitive means of identifying apoptotic cells, also did not reveal detectable levels of apoptosis in NG2 siRNA-treated cells (Fig. 3g). In contrast, phosphohistone H3-positive cells are reduced by 70 % in NG2 siRNA-treated pericytes (Fig. 3h–k), confirming a specific effect of NG2 ablation on cell proliferation.

Effects of NG2 ablation on pericyte migration were examined via transwell migration assays. Inclusion of PDGF-BB in the lower chamber was used to stimulate migration of pericytes transfected with NG2 siRNA and control siRNA species. Compared to large numbers of migratory pericytes in the control groups, pericytes transfected with NG2 siRNA exhibit markedly reduced migration (Fig. 3l–n). Quantification of cells on the lower side of the transwell membrane indicates that NG2 knockdown reduces PDGF-BB-induced pericyte motility by more than 60 % (Fig. 3o).

### NG2 knockdown reduces activation of β1 integrin and focal adhesion kinase signaling in pericytes

**Fig. 4:**
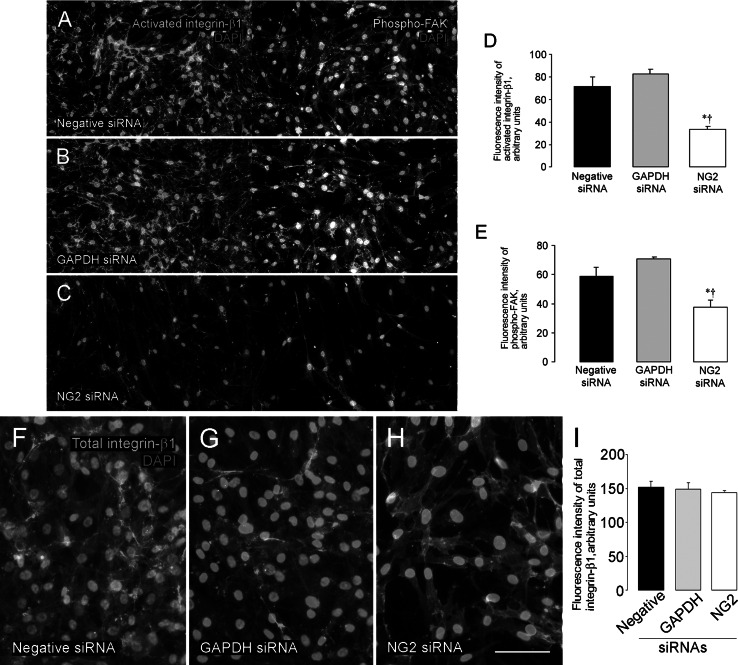
NG2 downregulation in pericytes decreases β1 integrin activation and downstream phosphorylation of focal adhesion kinase. To examine molecular mechanisms responsible for diminished pericyte function after NG2 downregulation, we examined activation of β1 integrin (mAb HUTS-21, *green*) and phosphorylation of its downstream target, focal adhesion kinase (P-Tyr371FAK, *red*). Activated β1 integrin and phosphorylated FAK are readily detectable in pericytes treated with negative siRNA (**a**) or GAPDH siRNA (**b**). However, treatment of pericytes with NG2 siRNA substantially reduces both β1 integrin activation and FAK phosphorylation (**c**), revealing the dependence on NG2 for activation of this signaling pathway. Measurements of fluorescence intensity demonstrate a 60 % reduction in activation of β1 integrin and a 40 % reduction in FAK phosphorylation (**d**, **e**). Total expression levels of β1 integrin (mAb TS2/16; green) in pericytes are not significantly reduced by treatment with any of the siRNA species (**f**–**i**). *Blue* = DAPI. *P < 0.05 versus negative siRNA, ^†^ *P* < 0.05 versus GAPDH siRNA. *Scale bar* 240 μm (**a**–**c**), 120 μm (**f**–**h**). (Color figure online)

The ability of NG2 to activate β1 integrin signaling when both molecules are expressed in the same cell [18, 20] suggests a mechanism for explaining the positive effect of NG2 on pericyte proliferation and motility. To investigate this possibility, we used the conformationally sensitive β1 integrin antibody HUTS-21 [19] to detect activation of β1 in human pericytes. We also used an antibody against phosphorylated focal adhesion kinase (phospho-FAK397) to detect FAK activation downstream of enhanced integrin signaling. In pericytes treated with control siRNA species, activated β1 integrin is abundant on pericyte cell surfaces (Fig. 4a, b). Phosphorylation of FAK is also robust in these control pericytes. However, NG2 siRNA-mediated knockdown of pericyte NG2 expression reduces levels of activated β1 integrin by 60 % (Fig. 4c, d). Phosphorylation of FAK in NG2-deficient pericytes is also reduced by 40 % compared to control pericytes (Fig. 4c, e). Compared to the significant reduction in activated β1 integrin levels in NG2 siRNA-treated pericytes, levels of total β1 integrin are not markedly changed after treatment with any of the siRNA species (Fig. 4f–i). These results support the idea that NG2-dependent activation of β1 integrin signaling (and subsequent phosphorylation of FAK) is a key mechanism underlying the ability of NG2 to promote pericyte proliferation and motility.

### NG2 knockdown in pericytes diminishes formation of pericyte/endothelial cell networks

**Fig. 5:**
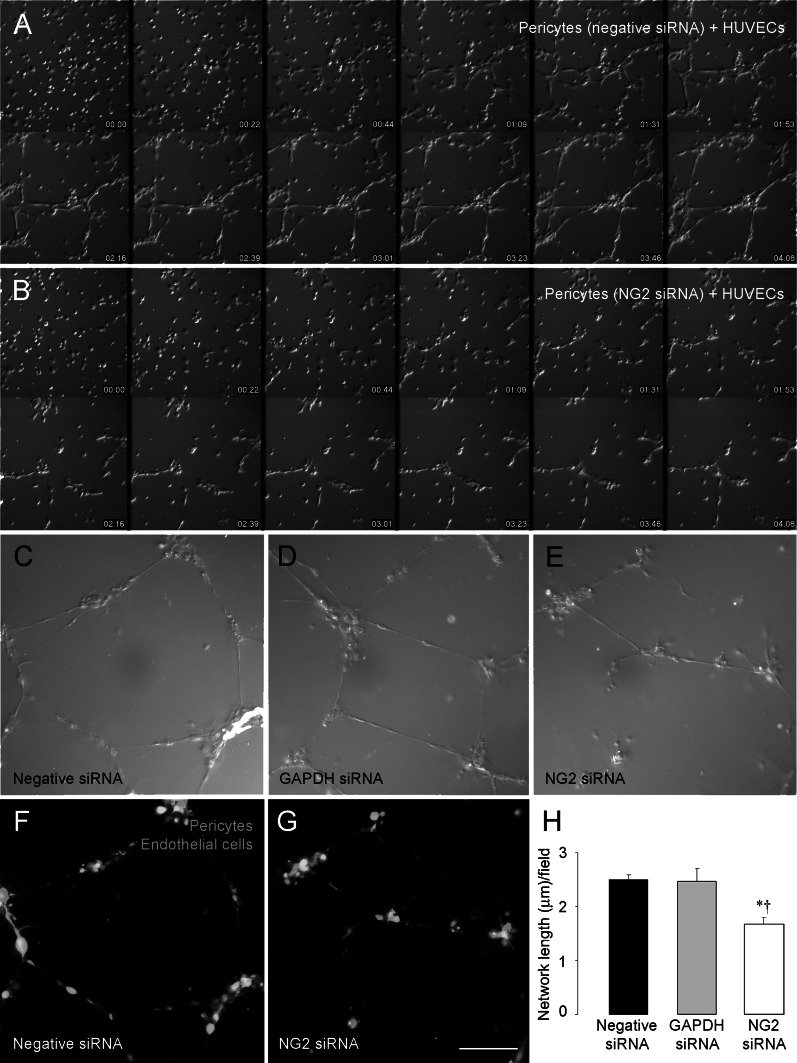
NG2 downregulation in pericytes retards formation of vascular networks with endothelial cells. Control pericytes and HUVECs were co-cultured at a 1:4 pericyte/HUVEC ratio in Matrigel. Initial signs of network formation are evident as soon as 1 h after the start of co-culture (**a**). Networks become increasingly organized between 2 and 4 h after co-culture. Network formation by NG2 siRNA-treated pericytes and HUVECs is significantly retarded at these same time points (**b**). Even after 8 h of co-culture, networks formed by NG2 siRNA-treated pericytes and HUVECs are less extensive than networks formed by control pericytes and HUVECs (**c**–**e**). Fluorescence images confirm that vascular networks at 8 h (**f**, **g**) are composed of both endothelial cells (labeled with CellTracker *Green* MFDA) and pericytes (labeled with CellTracker *Red* CMTPX). Networks containing NG2 knockdown pericytes (**g**) are again seen to be less extensive than those containing control pericytes (**f**). At 16 h of co-culture, quantification of total network lengths per unit area reveals that networks containing NG2 knockdown pericytes are 25 % less extensive than networks containing control pericytes (H). *P < 0.05 versus negative siRNA, ^†^ *P* < 0.05 versus GAPDH siRNA. *Scale bar* 480 μm (**a**–**b**), 240 μm (**c**–**g**). (Color figure online)

Since soluble, purified NG2 is effective in stimulating the formation of endothelial networks in vitro [9], we tested the effect of NG2 knockdown on the ability of pericytes to interact with endothelial cells to form pericyte/endothelial cell networks. Pericytes were treated with control or NG2 siRNA species, then co-cultured with HUVECs in Matrigel. Time-lapse imaging at early time points reveals the movement of both pericytes and HUVECs and the association of the two cell types to form three-dimensional networks in the Matrigel (Fig. 5). Three-dimensional networks containing both endothelial cells and control pericytes are evident after 2–4 h of co-culture (Fig. 5a; supplemental videoS1). In the case of NG2-deficient pericytes, however, network formation by pericytes and HUVECs is significantly retarded at these time points (Fig. 5b; supplemental videoS2). At 8 h, reduced network formation is still apparent in co-cultures of endothelial cells with NG2 knockdown pericytes (Fig. 5c–e). Use of CellTracker Red-labeled pericytes and CellTracker Green-labeled endothelial cells demonstrates the presence of both cell types in 8-h networks (Fig. 5f, g). Quantification of network formation at 16 h shows that the average total length of networks at 16 h is reduced 25 % by NG2 knockdown in pericytes (Fig. 5h).

### NG2 knockdown in pericytes reduces activation of β1 integrin in endothelial cells

**Fig. 6:**
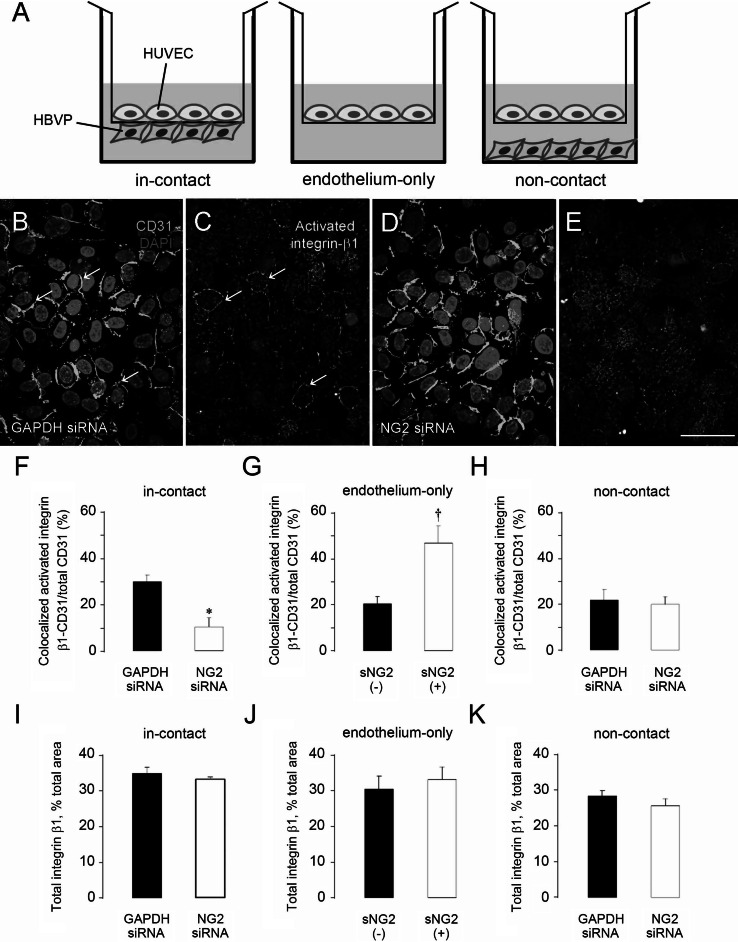
NG2 downregulation in pericytes decreases activation of β1 integrin in endothelial cells. **a** Three different in vitro culture formats were used to investigate interactions between human umbilical vein endothelial cells (HUVEC) and human brain vascular pericytes (HBVP). HUVECs and pericytes were cultured on opposite sides of a transwell membrane (in-contact); HUVECs alone were cultured on a transwell membrane (endothelium-only); HUVECs were cultured on a transwell membrane, and pericytes were cultured on the well bottom (non-contact). The in-contact model was used to examine pericyte-mediated activation of endothelial cell β1. Membranes were immunostained for CD31 (*green*) and activated β1 integrin (*red*), and confocal microscopy was used to examine the endothelial cell monolayer. CD31 labeling is similar in endothelial cell monolayers co-cultured with either control (**b**) or NG2 knockdown pericytes (**d**). However, activated β1 integrin is more prominent and more closely co-localized with CD31 on the surfaces of HUVECs co-cultured with control pericytes (**c**) than on HUVECs co-cultured with NG2 knockdown pericytes (**e**, quantified in **f**). In the endothelium-only model, β1 integrin activation is increased by the addition of soluble NG2 (sNG2) (**g**). In the non-contact model, activation of β1 integrin is not affected by treatments with control or NG2 siRNA (**h**). Total levels of β1 integrin expression are not affected by siRNA treatment in any of the three models (**i**–**k**). *P < 0.05 versus GAPDH siRNA (**f**). ^†^ *P* < 0.05 versus absence of sNG2 (**g**). *Scale bar* 120 μm (**b**–**e**). (Color figure online)

Soluble, purified NG2 can stimulate β1 integrin activation in endothelial cells [9]. We therefore examined whether pericyte cell surface NG2 can also activate β1 integrin signaling in endothelial cells as a means of altering endothelial cell morphogenesis. After treatment with the various siRNAs, pericytes were cultured on the lower surface of a transwell membrane with 0.4-μm-diameter pores. HUVECs were cultured on the upper surface of the same membrane (in-contact model in Fig. 6a). The membrane physically separates the two cell types and prevents cell migration across the membrane, but allows cell–cell contact via cellular processes that extend through the membrane pores [25–27]. After immunolabeling, confocal microscopy permits specific examination of the endothelial cell monolayer. Most HUVECs strongly express CD31 regardless of pericyte siRNA treatment, and in control-treated HUVECs activated integrin β1 integrin is often present on the cell surface along with CD31 (Fig. 6b, c, arrows). In contrast, after pericyte treatment with NG2 siRNA, levels of activated β1 integrin are reduced and are poorly co-localized with CD31 on cell surfaces (Fig. 6d–f).

We also used the transwell membrane system to re-examine β1 integrin activation in endothelial cells treated with purified, soluble NG2 (see Ref. [9]). Addition of soluble NG2 to an endothelial monolayer in an endothelium-only model (Fig. 6a) results in significant β1 integrin activation (Fig. 6g). This result suggests the possibility that NG2 shed from pericyte surfaces, rather than direct contact between pericytes and endothelial cells, might be responsible for the effect on β1 integrin activation seen in the double-monolayer model. This possibility was tested via use of a non-contact model in which endothelial cells were again grown on the upper surface of the transwell membrane, while pericytes were grown on the bottom of the transwells instead of on the lower surface of the membrane (Fig. 6a). In the absence of direct contact between pericytes and endothelial cells, knockdown of NG2 has no effect on β1 integrin activation in endothelial cells (Fig. 6h). Apparently, under these conditions, NG2 is not shed from pericytes in sufficient quantities to affect signaling in endothelial cells. Thus, in this format, direct contact between the two cell populations is required for NG2-mediated activation of β1 integrin signaling. As a control, we showed that there were no significant changes in expression levels of total β1 integrin in endothelial monolayers after treatment with any of the siRNA species or soluble NG2 (Fig. 6i–k).

### NG2 knockdown in pericytes reduces endothelial junctions and increases endothelial permeability

**Fig. 7:**
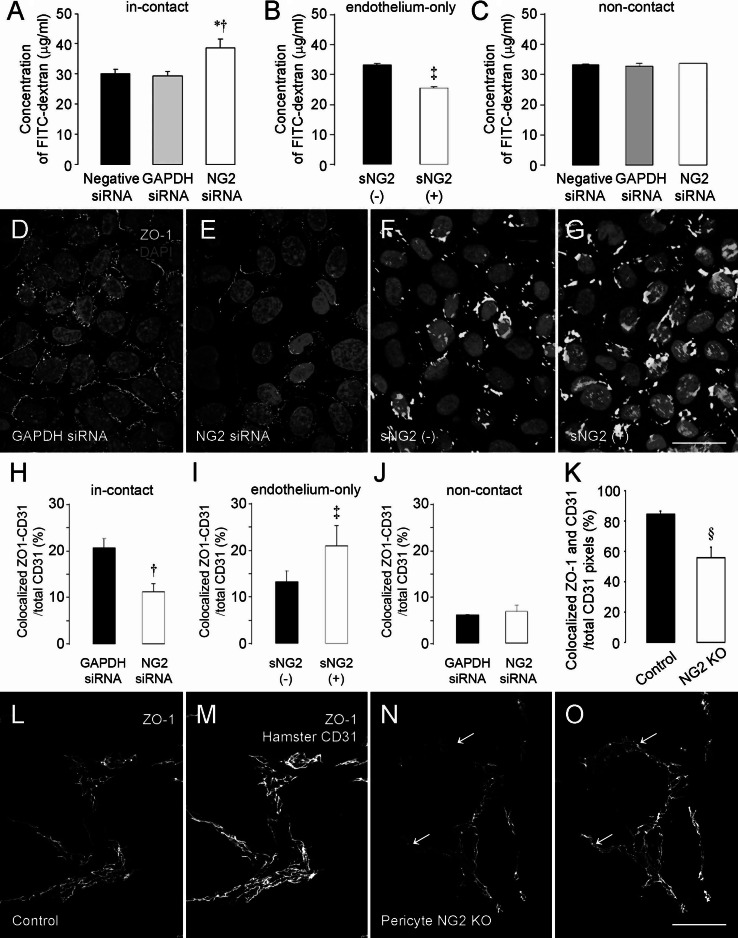
NG2 knockdown in pericytes reduces endothelial junction formation and barrier function. In the in-contact model, FITC-dextran leakage through endothelial monolayers co-cultured with NG2 knockdown pericyte monolayers is greater than leakage through endothelial monolayers co-cultured with control pericyte monolayers (**a**). Treatment with soluble NG2 (sNG2) significantly decreases FITC-dextran leakage through the endothelial cell monolayer in the endothelium-only model (**b**). In the non-contact model, NG2 knockdown in pericytes has no effect on endothelial permeability (**c**). Immunostaining for the endothelial cell junctional protein ZO-1 reveals extensive junction formation in endothelial monolayers co-cultured with control pericytes in the in-contact model (**d**). Junction formation is much less extensive in endothelial monolayers co-cultured with NG2 knockdown pericytes (**e**, quantified in **h**). Addition of soluble NG2 (sNG2) enhances junction formation between endothelial cells in the endothelium-only model (F,G, quantified in **i**). In the non-contact model, NG2 knockdown in pericytes does not affect endothelial cell junctions (**j**). Applying this same methodology to B16F10 tumor sections, we observed good correspondence between CD31 (*red*) and ZO-1 (*green*) immunolabeling in control mice (**l**, **m**). In contrast, portions of CD31-positive blood vessels in pericyte-NG2ko mice were deficient in staining for ZO-1 (**n**, **o** *arrows*; quantified in **k**). *Blue* = DAPI. *P < 0.05 versus negative siRNA (**a**), ^†^ *P* < 0.05 versus GAPDH siRNA (**a**, **h**). ^‡^ *P* < 0.05 versus absence of sNG2 (**b**, **i**). ^§^ *P* < 0.05 versus control mice (**k**). *Scale bar* 120 μm (**d**–**g**). *Scale bar* 60 μm (**l**–**o**). (Color figure online)

Since pericytes are important for stabilizing many properties of the endothelial lumen, we tested the ability of control and NG2-deficient pericytes to influence the permeability of endothelial monolayers. For this purpose, we again used transwell membranes with 0.4-μm-diameter pores, with endothelial monolayers grown on the upper surface and pericyte monolayers grown on the lower surface (in-contact model, Fig. 6a) [22, 23]. As in the β1 integrin activation experiments, contact between the two cell types is mediated by cellular processes that extend through the membrane pores. VEGF was included to stimulate permeability of the endothelial monolayer, and FITC-dextran was included in the upper chamber as a means of quantifying permeability. Leakage of FITC-dextran into the lower chamber through an endothelial cell monolayer associated with NG2 knockdown pericytes was increased by 32 % compared to leakage through an endothelial monolayer associated with control pericytes, demonstrating a decrease in barrier function (Fig. 7a). In the endothelium-only model (Fig. 6a), the permeability of the monolayer to FITC-dextran is decreased by 24 % after addition of soluble NG2, confirming the ability of NG2 to improve the barrier function of the endothelial monolayer (Fig. 7b). In the non-contact model (Fig. 6a), endothelial permeability is not affected by NG2 knockdown in pericytes (Fig. 7c).

To investigate the basis for the increased permeability of endothelial monolayers co-cultured with NG2-deficient pericytes in the in-contact model, we examined endothelial monolayers for the expression level and distribution of ZO-1, an important endothelial junction protein. Interestingly, the levels of ZO-1 expression and ZO-1 distribution around endothelial cells are significantly reduced in endothelial monolayers cultured with NG2-deficient pericytes (Fig. 7d, e, h), signifying a loss of endothelial junctions in the absence of pericyte NG2. Also in accord with results from the endothelial permeability studies, soluble NG2 significantly increases ZO-1 expression and co-localization with CD31 in the endothelium-only model (Fig. 7f, g, i). As expected, knockdown of NG2 in pericytes elicits no changes in ZO-1 expression or localization in the non-contact model (Fig. 7j). To determine whether NG2-negative tumor vessels in vivo are also characterized by loss of endothelial junctions, we examined ZO-1 expression in tumor blood vessels of control and pericyte-NG2ko mice (Fig. 7k–o). CD31 and ZO-1 localization are well matched in tumor vessels in control mice (Fig. 7l, m). However, ZO-1 labeling is absent from portions of CD31-positive tumor blood vessels in pericyte-NG2ko mice (Fig. 7n, o, arrows), resulting in a 35 % decrease in co-localization of ZO-1 and CD31 (Fig. 7k).

## Discussion

Throughout the formation, maturation, and maintenance of microvessels, pericytes and endothelial cells use a variety of mechanisms to communicate with each other. Endothelial cells produce PDGF-BB, which promotes pericyte recruitment via activation of PDGFRβ [28, 29]. Endothelial cell-derived TGFβ is also important for the recruitment and differentiation of pericytes [30, 31]. Pericytes produce angiopoietin-1, which activates Tie2 signaling to promote endothelial cell maturation [32, 33]. In addition, pericyte-derived vitronectin activates αv integrin signaling in endothelial cells to enhance endothelial survival via increased expression of the anti-apoptotic protein Bcl-w [34]. Adding to this list, we demonstrate in this report that pericyte expression of the NG2 proteoglycan is important not only for pericyte biology, but also for critical aspects of pericyte interaction with endothelial cells. Both of these NG2 functions appear to depend on its ability to activate β1 integrin signaling.

A number of previous studies in our laboratory have shown that NG2 promotes cell proliferation and motility in a variety of immature cell types [13]. Although NG2 is a transmembrane protein, it appears to have limited capability for independent signal transduction. Instead, via a physical interaction with β1 integrins [9, 18], NG2 facilitates integrin activation that leads to enhanced cell proliferation and motility [20]. The choice between proliferation and motility is determined at least in part by the phosphorylation status of the NG2 cytoplasmic domain. Protein kinase Cα-mediated phosphorylation at Thr-2256 results in localization of NG2/β1 integrin complexes to leading edge lamellipodia of glioma cells, accompanied by enhanced glioma cell motility. ERK-mediated phosphorylation at Thr-2314 results in localization of NG2/β1 integrin complexes to apical cell surface microprotrusions, accompanied by enhanced glioma cell proliferation [20]. Our current in vitro studies with human brain pericytes are consistent with this model of NG2-dependent β1 integrin activation. siRNA-mediated knockdown of NG2 expression in pericytes results in diminished activation of β1 signaling in the NG2-deficient pericytes, as revealed by labeling with the conformationally dependent β1 antibody HUTS-21 [19]. Decreased integrin signaling is accompanied by reduced downstream phosphorylation of FAK. Diminished signaling in this pathway is accompanied by decreased pericyte proliferation and motility. Although we did not examine pericyte proliferation or motility in vivo in the current work, we have previously reported in vivo evidence for reduced pericyte proliferation and motility in NG2 null mice. Reduced pericyte proliferation is observed in NG2 null mice in a retinal hyperoxia model [12] and in a corneal neovascularization model [35], leading to abnormal blood vessels in which endothelial cells are poorly ensheathed by pericytes. Reduced pericyte motility is seen in NG2 null mice in the corneal neovascularization model, in which NG2 ablation diminishes pericyte recruitment from limbal vessels to neovessels within the cornea [12].

In addition to functioning in a *cis* arrangement with both NG2 and β1 integrin expressed in the same cell, we suspected that NG2 might also be capable of operating in a *trans* mode to activate β1 integrin signaling in closely apposed cells. This is based on our observation that purified, soluble NG2 activates β1 signaling in endothelial cells in vitro, driving endothelial cell morphogenesis and the formation of vascular networks [9]. This expectation is borne out in our current work by the finding that NG2 knockdown in a pericyte monolayer reduces β1 integrin activation in an endothelial cell monolayer growing on the opposite face of a transwell membrane with 0.4-μm-diameter pores. A number of studies have demonstrated the ability of these membranes to prevent cell migration across the membrane while allowing cell–cell contact between processes that extend through the pores [25–27, 36, 37]. There are multiple consequences of reduced NG2-dependent β1 signaling in endothelial cells. The impact of pericytes on endothelial cell morphogenesis is decreased, as shown by the impaired interaction of pericytes with endothelial cells to generate complex vascular networks in vitro. Moreover, using the in-contact double-monolayer model on opposite sides of transwell membranes, we show that formation of endothelial junctions is reduced by NG2 knockdown in pericytes. This is evidenced by loss of expression/localization of the junctional molecule ZO-1. Accordingly, NG2 knockdown in pericytes reduces the barrier function of the endothelial cell monolayer, as revealed by increased leakage of FITC-dextran through the monolayer. The direct involvement of NG2 in improving the barrier function of the endothelial monolayer is confirmed by the ability of purified, soluble NG2 to decrease FITC-dextran leakage across the monolayer. However, NG2 does not appear to be shed by pericytes in sufficient quantities to affect endothelial cell properties in the in-contact double-monolayer model, since endothelial cell properties are not affected by pericytes grown with endothelial cells in a non-contact format. Thus, at least in these models, direct contact between pericytes and endothelial cells appears to be required for NG2-dependent activation of β1 integrin signaling and increased junction formation in endothelial cells.

Impaired interaction of NG2-negative pericytes with endothelial cells is also seen in our in vivo vascularization studies. Following exposure to hyperoxia, pathological blood vessels in the retina are poorly ensheathed by pericytes in the germline NG2 null mouse [12]. Germline ablation of NG2 also diminishes pericyte ensheathment of endothelial cells in both mammary tumors [10] and intracranial melanomas [11], leading to a number of vascular deficits, including decreased basal lamina assembly, impaired development of both pericytes and endothelial cells, decreased vessel patency, increased vessel leakiness, and increased intratumoral hypoxia. However, interpretation of these results has not been completely straightforward due to the global nature of the NG2 ablation in the germline knockout mice. In particular, NG2 is also ablated in myeloid cells, which are known to be important for tumor vascularization [38, 39]. The current study therefore uses pericyte-specific NG2 null mice in order to restrict NG2 ablation to the pericyte population.

Importantly, reduced pericyte ensheathment of endothelial cells is once again observed in melanoma tumor vessels in the pericyte-NG2ko mouse, demonstrating that this NG2-dependent deficit is pericyte autonomous. This decrease in pericyte/endothelial cell interaction leads to additional deficits in basal lamina assembly and endothelial junction formation, accompanied by decreased vessel patency, increased vessel leakiness, and increased intratumoral hypoxia. This is essentially the same spectrum of vascular defects observed in tumors in the germline NG2 null mouse, emphasizing the importance of NG2-mediated pericyte/endothelial cell interaction in determining the structural and functional properties of developing blood vessels. Nevertheless, it is noteworthy that vascular deficits detected in the pericyte-NG2ko mouse are generally less severe than those previously seen in the germline NG2 null mouse (Table 1). This suggests the possible contribution of other NG2-positive stromal cell populations to the overall process of tumor vascularization. Based on our immunohistochemical data, macrophages would appear to be the most likely NG2-positive candidate for this role. Future studies will examine the properties of tumor blood vessels in myeloid-specific NG2 null mice, which we are currently developing in our laboratory. Comparisons of tumor vascularization in pericyte-NG2ko and myeloid-NG2ko mice will reveal the relative vascular contributions of the NG2 expressed in these two cell populations.

**Table 1:** Vessel parameters in germline-NG2ko versus pericyte-NG2ko mice

Parameter	Change (compared to control mouse)	
	Global-NG2ko	Pericyte-NG2ko
Pericyte/endothelial overlap	45 % decrease	33 % decrease
Basal lamina assembly	73 % decrease	31 % decrease
Vessel patency	50 % decrease	43 % decrease
Vessel leakiness	400 % increase	270 % increase
Intratumoral hypoxia	2,000 % increase	560 % increase

Various parameters of tumor vessel structure and function are compared between the pericyte-NG2 ko mouse (this report) and the germline-NG2ko mouse [11]. Deficits in pericyte-NG2ko vessels are more moderate than changes in germline-NG2ko vessels

In conclusion, our data show that NG2 plays a dual role in pericyte biology, contributing not only to pericyte proliferation and motility, but also to pericyte interaction with endothelial cells. Interestingly, both roles of NG2 depend on the ability of the proteoglycan to activate β1 integrin signaling, either via a *cis* arrangement to activate pericyte proliferation/motility or via a *trans* arrangement to activate endothelial cell morphogenesis. These β1 integrin-dependent roles of NG2 appear to be very important for the formation of functional tumor vessels, as reflected by deficits in pericyte/endothelial cell interaction and in vessel function in pericyte-NG2ko mice.

## Electronic supplementary material

Below is the link to the electronic supplementary material.

Supplementary material 1 (AVI 41,477 kb)

Supplementary material 2 (AVI 41,477 kb)
